# Evaluating the Expression Levels of Human Endogenous Retrovirus-K 10 (HERV-K10) Gag as a Biomarker in Prostate Cancer Tissue

**DOI:** 10.7759/cureus.64275

**Published:** 2024-07-10

**Authors:** Imane Ouariagli, Soukayna Alaoui Sosse, Abdelilah Laraqui, Mohammed Reda Tagajdid, Mohammed Mrabti, Mohamed Alami, Moulay Mustapha Ennaji

**Affiliations:** 1 Laboratory of Virology, Oncology, Biosciences, Environment and New Energies, Faculty of Sciences and Techniques–Mohammedia, University of Hassan II Casablanca, Mohammedia, MAR; 2 Department of Urology, Mohammed V Military Teaching Hospital, University of Mohammed V Rabat, Rabat, MAR

**Keywords:** tissue, qrt-pcr, prostate cancer, biomarker, hervk-10

## Abstract

Prostate cancer is one of the most common major health problems. Several risk factors are potentially involved in its development. Therefore, a biomarker capable of early diagnosis is necessary to facilitate the early detection and treatment of prostate cancer. Human endogenous retroviruses (HERVs) are abnormally expressed in various diseases. Our study aims to evaluate the specific role of HERV K-10 gag expressions in the progression of prostate cancer. For this, we collected a set of 50 prostate tumor tissue samples as well as 50 healthy tissue samples. After extracting RNA from the prostate samples, we analyzed the expression of HERV-K gag using quantitative real-time PCR (qRT-PCR). The resulting data revealed a significant correlation of HERV-K gag expression in malignant regions of the prostate in men with prostate cancer than in men without prostate cancer (p < 0.05). The presence of the HERV-K gag protein was detected in 10 of 50 tumor samples (20%), while no healthy samples presented this protein. These results suggest that increased HERV-K gag RNA and protein expression could serve as a sensitive and specific biomarker of prostate malignancy in this cohort of prostate carcinoma patients, further supporting its potential as a promising clinical marker.

## Introduction

Prostate cancer is one of the most common cancers in men globally, representing a major public health challenge [1]. Despite advances in diagnostic and treatment methods, the clinical variability of this disease remains a major obstacle in patient care. In this context, the search for early and reliable biomarkers of tumor progression is of crucial importance [2].

Known risk factors for prostate cancer include older age, family history of prostate cancer, race, and environmental factors such as diet and lifestyle [3].

Human endogenous retroviruses (HERVs) are genomic elements derived from past viral infections that have become integrated into the human genome through evolution. Among HERVs, the HERV-K subgroup has been particularly studied due to its possible involvement in the development of various cancers. HERV-K gag is a protein encoded by this class of retroviruses [4].

Previous studies have suggested a correlation between abnormal HERV-K gag expression and the progression of various cancers, including prostate cancer. This led to the hypothesis that HERV-K gag may play a role in carcinogenesis by modulating cellular processes such as proliferation, invasion, and metastasis [5].

The objective of this study was to evaluate the role of HERV-K gag expression in the progression of prostate cancer. We seek to determine whether HERV-K gag expression is associated with prostatic malignancy and whether it could serve as a potential biomarker for the diagnosis, prognosis, and therapeutic management of this disease. Using prostate tissue samples from prostate cancer patients, we will analyze HERV-K gag expression and evaluate its correlation with tumor progression, with the aim of better understanding the underlying mechanisms of this disease and identifying new potential therapeutic targets.

## Materials and methods

Samples

The study collected 50 samples from prostate cancer patients from the Mohammed V Military Hospital in Rabat, Morocco, and 50 normal samples from a private medical laboratory. Patient population demographics were collected from medical records and are summarized in the Results section of this article. All experiments were carried out in accordance with the standards of the Casablanca Biomedical Research Ethics Committee (n°3/2018), and written informed consent was obtained from all recruited patients.

RNA extraction

To extract RNA from patient samples, we meticulously followed the manufacturer's protocol. Initially, all tissues were suspended in 1 mL of RNX-plus solution reagent (Cinnagen, Tehran, Iran) using a homogenizer. Subsequently, chloroform was added, followed by centrifugation to extract the protein from the solution. Isopropanol was then added to precipitate RNA from the supernatant. The resulting RNA solution was diluted with 50 μL of treated water and treated with DNase enzyme to eliminate genomic DNA contamination. Verification of RNA purity was performed using conventional PCR to confirm the absence of genomic DNA contamination. The final RNA product was stored at -80°C in RNase-free tubes. Quality assessment of the extracted RNA was conducted using a nanodrop device.

In this study, two primers (forward and reverse) were designed for the HERV-K gag gene. The β-globin gene primers were also designed as primers of a reference gene. The primers of HERV-K gag and β-globin are shown in Table 1.

**Table 1:** Primer sequences of HERVK gag and β-globin

Oligonucleotide	Sequences of primers
β-globin	Forward 5'-CAACTTCATCCACGTTCACC-3' Reverse 5’-GAAGAGCCAAGGACAGGTAC-3’
HERVK gag	Forward 5’-TAATACGACTCACTATAGGAACAGACCACCATGGGGCAAACTAAAAGT-3’ Reverse 5’-CAGGCAGTGGGCCATATAC-3’

cDNA synthesis

The reverse transcription process was conducted using the Bio Fact cDNA kit (Daejeon, South Korea). To facilitate the conversion of RNA to DNA, a 20 µL reaction mixture was prepared, consisting of 1 µL of random hexamer, 9 µL of master mix, and 10 µL of RNA samples. Incubation ensued for 40 minutes at 50°C, followed by a subsequent 10-minute period at 95°C within a Bio Intellectica PCR machine. Following synthesis, the resulting cDNA was subjected to a twofold dilution using sterile water.

Quantitative real-time PCR

In this method, we evaluated changes in mRNA expression levels using the HyperScript kit (Gene All, South Korea) according to the manufacturer's instructions.

Reactions were carried out in a total volume of 20 μL, containing 10 μL of master mix, 6 μL of sterile water, 1 μL of 10 pmol forward primer, 1 μL of 10 pmol reverse primer, and 2 μL of cDNA.

Cycling conditions were as follows: one cycle at 95°C for 10 minutes, 40 cycles at 95°C for 30 seconds, 55°C for 30 seconds, and 72°C for 30 seconds in a Rotor-Gene 6000 (Corbett Life Sciences, Sydney, Australia) in 36-well gene disks. The melting curve ranged from 60°C to 95°C. The housekeeping gene GAPDH was amplified as an internal control, and relative quantification values were calculated based on a 2-ΔΔct expression formula.

Statistical analysis

JAMOVI 23.0 statistical software was used to evaluate the potential association between various disease parameters and the presence of HERV-K. Statistical significance was determined based on a p-value less than 0.05.

## Results

**Figure 1:**
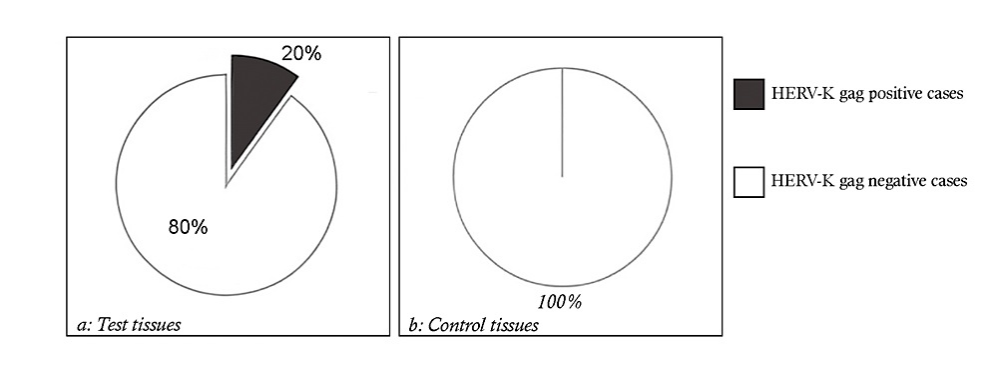
HERV-K gag mRNA expression in prostate cancer tissues and control samples: (a) percentage of HERV-K gag positive and negative cases in tissues; (b) percentage of HERV-K gag positive and negative cases in control samples This figure illustrates the results of the analysis of HERV-K gag mRNA expression in prostate cancer tissues and control samples. The graphs show the percentage of samples testing positive and negative for HERV-K gag mRNA expression in both groups. In the prostate cancer tissue samples, 20% tested positive for HERV-K gag mRNA expression, while 80% tested negative. In contrast, all control tissue samples tested negative for HERV-K gag mRNA expression. This figure highlights the potential association between HERV-K gag expression and prostate cancer, suggesting its relevance as a biomarker for the disease.

The concentration of the RNA extract was verified using a nano-drop apparatus, where the ratio of 260 to 280 was found to be within the expected range of 1.8 to 2.2. Additionally, the 260 to 230 ratio for RNA was determined to be greater than 2, consistent with our findings. Furthermore, RNA extracted from PCR amplification was analyzed using a 2% agarose gel to assess the quality of the process, and the results were photographed. No contamination by genomic DNA at the RNA level was observed. HERV-K gag mRNA expression showed that in 10 (20%) out of 50 samples from prostate cancer tissues, the RT-PCR test was positive, while in the remaining samples (80%), the RT-PCR test was negative. However, evaluation of 50 control tissue samples by RT-PCR revealed no samples in 50 (0%) (Figure 1).

The expression of the HERV-K gag gene in the sample suggests that it is more likely to be expressed during cancer or disease progression. The resulting data revealed a significant correlation of HERV-K gag expression in malignant regions of the prostate in men with prostate cancer compared to men without prostate cancer (p < 0.05) (Table 2).

**Table 2:** Correlation between HERV-K gag gene expression and prostate cancer progression *p < 0.05 is considered statistically significant.

HERV-K gag gene expression	Expressed (positive)	Non-expressed (negative)	P-value
Case group (n=50)	10 (20%)	40 (80%)	0.0016*
Control group (n=50)	0 (0%)	50 (100%)	0.033

## Discussion

Prostate cancer remains a major health problem, requiring the development of reliable biomarkers for early diagnosis and treatment [6]. Many factors can lead to prostate cancer, including age, geographic location, genetic mutations, and infections [7]. For almost thirty years, various studies have examined the role of viral infections in the development and spread of cancer [8]. A group of viruses that have recently emerged due to their association with cancer is the HERV family, remnants of ancient germ cell infections that are now part of the human genome. Retroviral HERV-K elements are inhibited due to their presence in heterochromatin fragments and their antiviral defense mechanisms. In the case of cancer, these retrotransposons will emerge from a dormant state and resume activity [9].

In our present study, we aimed to evaluate the expression of the HERV-K gag gene and its potential role as a biomarker for prostate cancer. We found a significant correlation between HERV-K gag expression and prostate malignancy, highlighting its potential utility in clinical diagnosis.

These results are consistent with previous research indicating the expression of HERVs in various cancers. One study detected HERV-K gag protein in 12 of 18 (66.7%) malignant tissues, but only in 1 of 18 (5.6%) benign tissue sections. HERV-K gag expression was significantly higher in malignant regions of the prostate in men with prostate cancer than in matched benign regions and in men without prostate cancer (p < 0.001 for both) [10]. Likewise, a study by Reis et al. revealed that anti-GAG-HERV-K antibodies are detected in a subgroup of prostate cancer patients (33 of 483, 6.8%), but rarely in male donors in good health (1 in 55, 1.8%) [11].

Specifically related to prostate cancer, our study aligns with the work of Wallace et al., who found significantly higher levels of HERV-K RNA in prostate cancer tissues compared to benign prostatic hyperplasia. Their research supports our observation that HERV-K gag RNA and protein expression are increased in malignant prostate tissues. The detection of HERV-K gag protein in 20% of our tumor samples, with no presence in healthy samples, reinforces the potential of HERV-K as a specific biomarker for prostate cancer [12]. Other studies on groups of patients and control groups showed that the increase in mRNA expression was positive in 64% of patients with breast cancer and negative in all healthy people [13]. The resulting data from a similar study revealed that although there was a significant increase in the expression level of HERV-K gag and np9 in breast cancer tissues (P ≤ 0.01, 0.05, 0.05, respectively), we found no significant elevation in expression rec mRNA level [14].

The significant expression of HERV-K gag in malignant prostate tissues, as shown by our qRT-PCR results, highlights its potential as a sensitive and specific biomarker for prostate cancer. This is particularly important for early detection, where traditional biomarkers such as PSA (prostate-specific antigen) may have limitations due to false positives and lack of specificity. Integrating analysis of HERV-K gag expression could improve diagnostic accuracy, allowing better differentiation between malignant and benign prostate conditions.

Although our study provides compelling evidence for the role of HERV-K gag in prostate cancer, it is essential to recognize some limitations. The sample size, although sufficient for preliminary analysis, should be increased in future studies to validate these results in a larger and more diverse cohort. Additionally, further research should explore the underlying mechanisms by which HERV-K influences tumorigenesis and its interaction with other oncogenic pathways.

Future studies should also investigate the potential of HERV-K gag as a therapeutic target. Given its specific expression in malignant tissues, HERV-K gag could be explored for targeted therapies, possibly involving immune modulation or direct inhibition of its expression.

## Conclusions

Our study provides significant insights into the role of HERV-K gag in prostate cancer, supporting its potential as a promising biomarker for early detection and diagnosis. The correlation between HERV-K gag expression and prostate malignancy aligns with existing research on HERVs in other cancers, reinforcing the importance of this biomarker in clinical practice. Further research is needed to validate these results and explore the HERV-K gag target's therapeutic potential in prostate cancer treatment.
